# A case report of fibrolamellar hepatocellular carcinoma, with particular reference to preoperative diagnosis, value of molecular genetic diagnosis, and cell origin

**DOI:** 10.1186/s40792-021-01295-4

**Published:** 2021-09-17

**Authors:** Atsushi Takahashi, Hiroshi Imamura, Ryota Ito, Fumihiro Kawano, Yu Gyoda, Hirofumi Ichida, Ryuji Yoshioka, Yoshihiro Mise, Yuki Fukumura, Katsuhiro Sano, Akio Saiura

**Affiliations:** 1 Department of Hepatobiliary-Pancreatic Surgery, Juntendo University School of Medicine, Juntendo University Hospital, 3-1-1 Hongo, Bunkyo-Ku, Tokyo, 113-0033 Japan; 2 Department of Pathology, Juntendo University School of Medicine, Tokyo, Japan; 3 Department of Radiology, Juntendo University School of Medicine, Tokyo, Japan

**Keywords:** Fibrolamellar hepatocellular carcinoma, DCP, *DNAJB1*-*PRKACA*

## Abstract

**Background:**

Fibrolamellar hepatocellular carcinoma (FL-HCC) is a liver tumor that occurs almost exclusively in young adults without underlying liver disease. In spite of its distinct clinical characteristics and specific imaging findings, preoperative diagnosis is often difficult due to the extremely low incidence of the tumor. Although FL-HCC shows particular morphological features on H&E-stained tissue sections, differential diagnosis from ordinary HCC, especially the scirrhous variant of HCC, and intrahepatic cholangiocarcinoma needs additional immunohistochemical (IHC) analyses and/or molecular genetic testing.

**Case presentation:**

A 21-year-old male patient was referred to our hospital for further evaluation of a large liver mass. Abdominal ultrasound examination, contrast-enhanced computed tomography, and magnetic resonance imaging revealed a well-defined hypervascular lobulated liver mass, 11 × 11 cm in diameter, with a central scar and calcification, in segments 5/8. Under the diagnosis of FL-HCC, we carried out extended anterior sectorectomy, including a part of segment 4. On microscopic examination, the tumor was composed of proliferating polygonal cells with abundant eosinophilic granular cytoplasm containing nuclei with vesicular chromatin and enlarged nucleoli, in an abundant stroma. Collagen fibers arranged in a parallel lamellar pattern were seen in the tumor stroma. These findings, together with the results of subsequent IHC analyses using HAS, CK7, and CD 67, we made the diagnosis of FL-HCC, which was further confirmed by detection of the *DNAJB1*-*PRKACA* fusion gene in the tumor cells by RT-PCR.

**Conclusion:**

FL-HCC shows distinct imaging appearances. Although it also has characteristic morphological features, combined use of IHC and/or molecular genetic studies are necessary for the final diagnosis.

## Background

Fibrolamellar hepatocellular carcinoma (FL-HCC) was first described in 1956 by Edmondson [1], and named as FL-HCC by Craig in 1980 [2]; however, it was not until 2010 that FL-HCC was assigned its own WHO classification code [3]. Despite its distinct clinical characteristics, i.e., predominant occurrence in adolescents and young adults (ages between 5 and 40 years) who do not have underlying liver disease [4], preoperative diagnosis of FL-HCC is often difficult because of its extremely low incidence, with an estimated age-adjusted incidence rate of 0.02 per 100,000 in the United States [5]. This aspect becomes further emphasized in countries including Japan, where the prevalence of ordinary hepatocellular carcinoma (HCC) is relatively high. Herein, we present the case of a 21-year-old man, who we diagnosed preoperatively as having FL-HCC based on the findings of abdominal ultrasound (US), CT, and MRI. The diagnosis was confirmed by histological assessments, including immunohistochemical (IHC) analysis, and further validated by the detection of the *DNAJB1*-*PRKACA* fusion gene by RT-PCR.

## Case presentation

A 21-year-old Japanese male patient visited a neighborhood hospital with a history of fever (38℃). He was diagnosed as having a large liver mass by US of the abdomen and was referred to our hospital. There was no history of body weight loss, abdominal pain, nausea, or jaundice. There was no significant past medical history, including of liver disease and/or blood transfusion. Laboratory investigations revealed the following: percent prothrombin time, 84.0%; albumin, 4.2 g/dl; total bilirubin, 0.85 mg/dl; AST, 52 IU/l; ALT, 100 IU/l; ALP, 514 IU/l; γ-GTP, 150 IU/l; and cholinesterase, 420 IU/l. Tests for HBs Ag and HCV Ab were negative. Tests for tumor markers revealed elevation of the plasma level of des-gamma carboxyprothrombin (DCP; 8768 mAU/mL); however, the serum levels of other tumor markers that might have indicated the pathological nature of the liver mass, i.e., AFP, AFP-L3, CEA, and CA19-9, were as follows: AFP, 2 ng/ml; AFP-L3, 0.5%; CEA, 2.1 ng/ml; and CA19-9, 10 U/ml, which were all within their respective normal ranges. Abdominal US performed at our hospital revealed a heterogeneously hyperechoic liver mass measuring 11 cm in diameter, with a central linear band over the anterior sector.

**Fig. 1:**
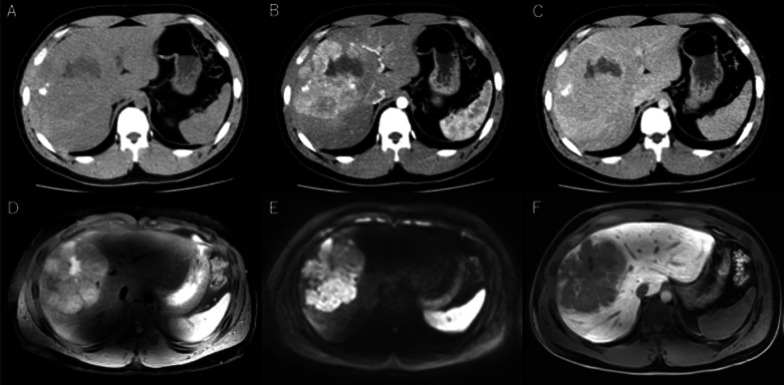
Dynamic computed tomography (CT) and gadoxetic acid-enhanced magnetic resonance imaging. A lobulated mass is visualized located in the anterior sector of the liver. The tumor was seen as a low-attenuating lesion in unenhanced images (**A**) and as a heterogeneous hyperattenuation in the arterial phase of dynamic CT (**B**). Hyperattenuation in the arterial phase suggests hypervascularity. The tumor demonstrated washout in the delayed phase of dynamic CT (**C**). The central scar showed a gradually enhancing pattern with calcification. The scar was seen as a hypointensity on T2WIs (**D**) and as a marked hyperintensity on DWIs (**E**). The tumor was visualized as a low signal intensity in the hepatobiliary phase, without gadoxetic acid uptake. Also, the tumor was observed as having a lobulated appearance in the hepatobiliary phase (**F**)

Contrast-enhanced computed tomography (CT) showed a well-defined lobulated liver mass, 11 × 11 cm in diameter, in the anterior sector, with a central unenhanced low-density area. Calcification was observed inside the mass. In the arterial phase, the tumor showed heterogeneous enhancement, while in the delayed phase, a large part of the tumor was detected as an iso-density area, leaving the slightly enhanced central part, which was suggestive of the presence of a central scar (Fig. 1A–C). On magnetic resonance imaging (MRI), the mass was identified as a hypointense tumor not containing lipid in T1-weighted images, as well as during the subsequent opposed phase; while in T2-weighted image, the lesion was visualized as a slightly hyperintense tumor with a focal hypointense area representing the central scar. In the hepatobiliary phase of gadoxetic acid-enhanced MRI, the tumor was visualized as a hypointensity as compared to the surrounding liver tissue (Fig. 1D–F). On PET–CT, the main lesion was clearly visualized in the maximum intensity projection (SUVmax = 4.67). Accumulation was also detected in a hepatoduodenal lymph node (SUVmax = 4.31), suggestive of metastasis.

The tumor occupied the anterior sector and partially extended to segment 4, but did not contact with posterior glissonean branch or right hepatic vein. After confirming a normal ICG R15 value (3.5%) and an acceptable estimated future remnant liver volume (64%), we carried out an extended anterior sectorectomy (segments 5 and 8), including a part of segment 4. The hepatoduodenal lymph nodes were also resected, but were revealed to be negative for cancer by rapid histopathological diagnosis during surgery. The operation time was 427 min and the blood loss was 1152 ml.

**Fig. 2:**
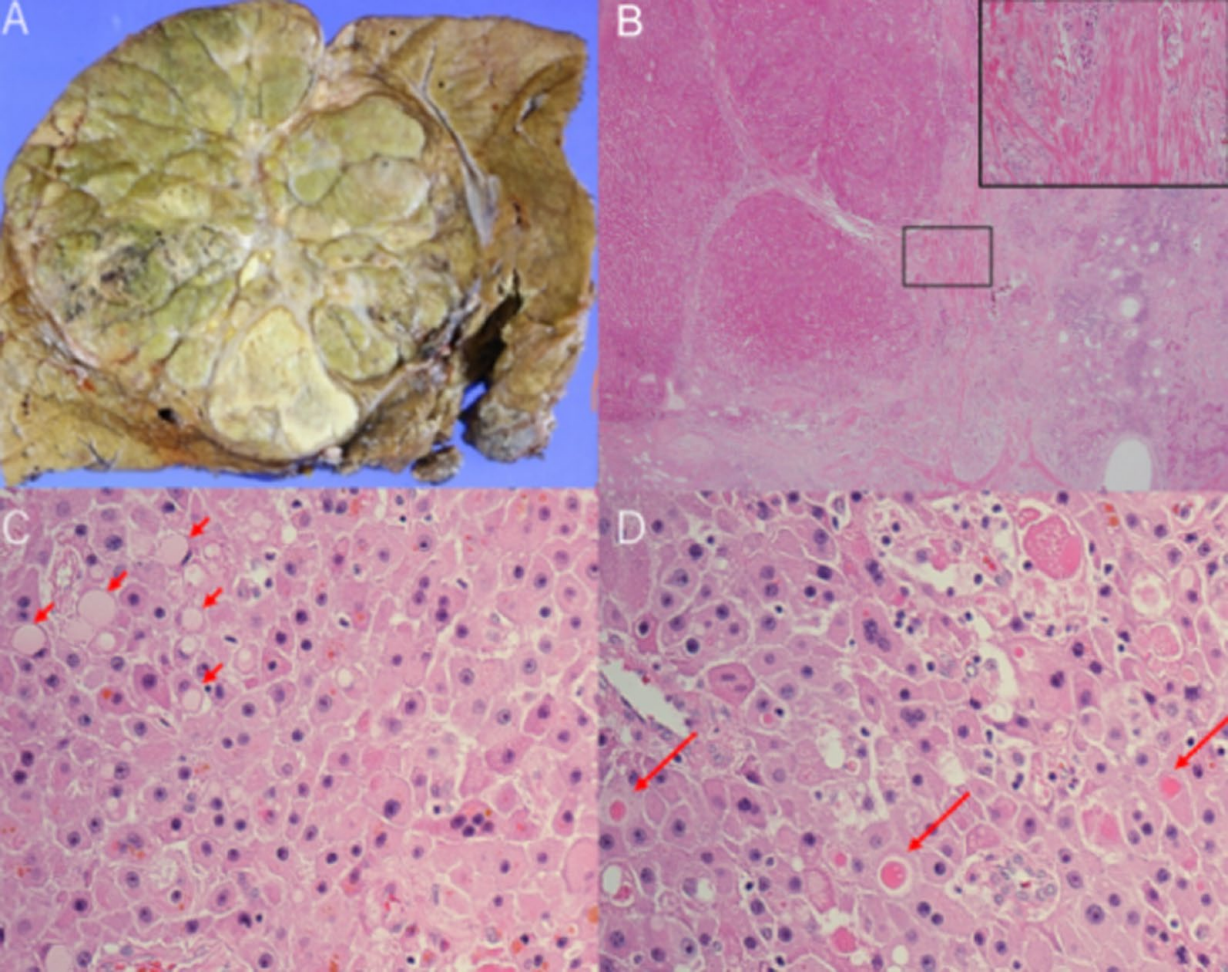
Pathological view of the tumor. The tumor was a well-defined lobulated tumor with a central scar on gross examination (**A**). The central portion of the tumor showed abundant fibrous stroma in a lamellar pattern (inset) containing tumor cells. (H&E, × 100) (**B**). Tumor cells had large nuclei and often contained pale bodies (short arrows) (H&E, × 400) (**C**). Intracellular hyaline globules were often observed as well (long arrows, × 400) (**D**)

**Fig. 3:**
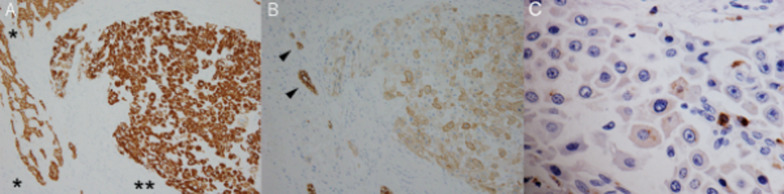
Immunohistochemical findings of the tumor. Tumor cells showed diffuse, intense staining for Hep Par-1. Note that more intense staining for the tumor part (**) compared to the background hepatic parenchyma (*) (**A**). Tumor cells showed diffuse, weak staining for CK7. Arrowheads indicate bile ductules (**B**). Positive staining for CD68 was also noted, although it was only focal staining (**C**)

**Fig. 4:**
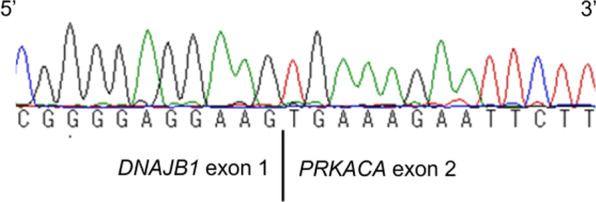
With RT-PCR using FFPE tissue, *DNAJB1-PRKACA* fusion gene was identified

On gross examination, a tumor measuring 11 × 11 cm in size containing a central scar was seen. The tumor was composed of multiple greenish or yellowish nodules, with partially hemorrhagic and necrotic areas. Multiple intrahepatic metastatic nodules were also detected. The surgical margin was free from tumor. The background liver structure was unremarkable. Microscopically, proliferating tumor cells containing abundant eosinophilic granular cytoplasm with nuclei containing enlarged nucleoli were seen with an abundant stroma. Intracytoplasmic inclusion bodies, that is, pale bodies, as well as hyaline globules were frequently detected. Collagen fibers arranged in a parallel lamellar pattern was seen in the tumor stroma. Occasional vascular invasion by the tumor was also identified. The background liver was normal (Fig. 2A–D). These findings, together with the particular background characteristics, i.e., the patient being a young adult without underlying liver disease, were highly suggestive of the diagnosis of FL-HCC. Immunohistochemically, the tumor cells were diffuse, intense positive staining for both Hep Par-1 (clone OCH1E5, DAKO, Denmark) and CK7 (clone OV-TL 12/30a, Leica Biosystems, Newcastle upon Tyne, UK). Positive staining for CD68 (clone KP-1, Abcam, Cambridge, UK) was also observed, but only focally (Fig. 3). Based on these histopathological and IHC findings, the tumor was diagnosed as a FL-HCC. To further confirm this diagnosis, the presence of *DNAJB1*-*PRKACA* fusion transcript (*DNAJB1* exon 1 and *PRKACA* exon 2) was examined; five sections, each 4- to 5-μm thick, were prepared from the representative paraffin-embedded tumor specimen. Following dewaxation/dehydration of the specimen, total RNA was extracted utilizing RNeasy formalin fixed paraffin embedded (FFPE) Kit (Qiagen, Hilden, Germany) according to the manufacturer's instructions. Then, the presence of the fusion product was investigated by RT-PCR using SuperScript™ VILO™ cDNA Synthesis Kit (Thermo Fisher Scientific, AL, USA) and using primer pairs designed using Primer3 PLUS software prepared by Untergasser et al. [6], referring to the previous report [7]. The obtained PCR product was sent for the sequence analysis after purification and the presence of *DNAJB1*-*PRKACA* fusion transcript was confirmed (Fig. 4).

The patient’s postoperative course was uneventful, and the plasma DCP level decreased to the normal range almost immediately. The patient was discharged from the hospital on postoperative day 25. He has been under follow-up at our outpatient clinic, without showing evidence of recurrence until now, 17 months since the surgery.

## Discussion

While FL-HCC almost exclusively affects adolescents and young adults who do not have underlying liver disease [4], preoperative diagnosis of FL-HCC is not always easy, because of its extremely low incidence. Indeed, non-cirrhotic HCC is still more common in the young adult population than FL-HCC [5, 8, 9]. From the viewpoint of preoperative imaging studies, the differential diagnosis of a large hepatic tumor(s) would include FL-HCC, HCC, hepatocellular adenoma, and focal nodular hyperplasia (FNH) [10–14]. Considering that both HCC and FL-HCC are definite indications for surgery, and that surgery is also often indicated for large hepatocellular adenomas because of the high probability of the presence of concomitant carcinoma, the most important differential diagnosis that should be ruled out is FNH. Several differences in the imaging findings between FL-HCC and FNH have been reported. First, in the majority of cases, FNHs are round in shape [15, 16]. Second, on T2-weighted MR images, a central scar is visualized as a high signal intensity in the case of FNH [15, 16]. The scar in cases of FNH rarely shows calcification on CT images [15]. Third, homogeneous hyperattenuation is seen in the arterial-phase images of dynamic CT and/or MRI in the case of FNH [15–17]. Fourth, FNH shows a lower signal intensity and higher apparent diffusion coefficient (ADC) than FL-HCC on diffusion-weighted imaging (DWI) in MRI [18–21]. Finally, FNH is visualized as an iso- to hyperintensity in comparison with the surrounding normal liver tissue in the hepatobiliary phase of gadoxetic acid-enhanced MRI [17, 20, 22, 23]. The most important findings of differentiation from FNH are the low signal intensity in the hepatobiliary phase of gadoxetic acid-enhanced MRI. All of these features were applicable to the diagnosis in our patient reported herein.

Histologically, this case showed typical histology, such as large polygonal tumor cells containing abundant eosinophilic granular cytoplasm, large nuclei and nucleoli [3, 24, 25], pale bodies and pink bodies, intratumoral fibrosis, often in a parallel pattern [3, 24, 25]. Although these findings are suggestive for FL-HCC, they are not definitive, since all of the four aforementioned features can also be found in cases of ordinary HCC or scirrhous variant of HCC (scirrhous HCC) [25]. Although our IHC assessment further supported the diagnosis of FL-HCC [26–28], we considered the genetic support is also necessary for the definite diagnosis, since some HCC and its variants can also show Hep Par-1 and CK7 positive. Regarding CD68 (clone KP1)-IHC, its positivity has been reported helpful in differentiating FL-HCC from conventional HCC [29], where CD68-positivity suggests the abundance of intracellular lysosomes, characteristic feature of FL-HCC. However, only a few tumor cells were positive for CD68 in the present case, and we consider that further IHC and electron microscopic studies may be warranted to see the significance of CD68-IHC in FL-HCC.

In 2014, Honeyman et al. described a *DNAJB1*-*PRKACA* fusion transcript that resulted in overexpression/activation of protein kinase A in all 10 specimens of FL-HCC examined [30]. This seminal discovery paved the way for molecular-based diagnosis of FL-HCC, as well as the pathogenic role of this fusion transcript. Although subsequent investigations have consistently shown that the *DNAJB1*-*PRKACA* transcript or the resultant fusion is 100% specific for FL-HCC [31], one multi-institutional study alone reported that 10–20% of FL-HCC specimens examined were negative for this fusion transcript [32]. However, this could be attributable to the pathological examination for FL-HCC having been performed independently at each participating institution separately, possibly leading to the unreliability of a bona fide diagnosis. Indeed, in another study comprising 124 patients from 13 institutions worldwide who were initially classified as having FL-HCC [25], a central review by two pathologists confirmed typical FL-HCC in 104 cases, whereas the remaining cases were re-classified as “possible FL-HCC” (12 cases) or “unlikely to be FL-HCC” (8 cases). Subsequent molecular testing revealed a positive result for the *DNAJB1*-*PRKACA* fusion transcript in 103 of the 104 typical FL-HCC cases, with the remaining one case with the Carney complex characterized by germline *PRKAR1A* mutations [33]. In contrast, only 9 of the 12 cases of “possible FL-HCC” and none of the 8 cases of “unlikely to be FL-HCC” showed a positive result for the fusion transcript. Hence, it can be reasonably concluded that the molecular testing for the *DNAJB1*-*PRKACA* fusion transcript is a 100% sensitive/specific diagnostic test for FL-HCC, except for the very rare cases of complete loss of *PRKAR1A*, i.e., the Carney complex [34, 35].

One of the unresolved questions in FL-HCC is its cell origin. As seen in this case, H&E-stained morphology is similar to ordinary HCCs. Although IHC suggested both hepatocellular differentiation (Hep Par-1 positivity) and cholangiocytic differentiation (CK7 positivity) [27], pathological impression may be hepatocytic origin, since CK7-positivity is infrequently seen for moderately to poorly differentiated conventional HCCs. Several investigators have hypothesized a liver stem cell origin of FL-HCC, because of the frequent gross vascular invasion [36] and lymph node metastases [2, 27], clinical phenotypes that are more frequently associated with HCC and ICC, respectively. Oikawa et al. reported that FL-HCCs were most similar to biliary stem cells as compared to the three other maturational stages of the liver, i.e., hepatic stem cells, hepatoblasts, and human adult hepatocytes, based on the gene expression profile [37]. In their subsequent study with the Cancer Genome Atlas [8], however, FL-HCC did not cluster with ICC, which are commonly thought to be derived from normal biliary cells. A phenotypic aspect that has not received sufficient attention is the tumor marker profiles. Although FL-HCC is frequently detected at an advanced stage as a large liver tumor(s), the serum AFP level, as also other markers of adenocarcinoma, such as CEA and/or CA19-9, is reportedly within normal range [24, 27, 31]. By contrast, the majority of patients with FL-HCC, including our patient reported herein, show abnormal plasma levels of DCP, a marker exclusively found in cases of HCC [36]. This characteristic favors the assumption that HCC and FL-HCC share a common origin.

## Conclusion

We have described the case of a patient with FL-HCC, in whom the tumor was diagnosed preoperatively by various imaging modalities. The diagnosis was confirmed by histopathological examinations, including light microscopy of H&E-stained sections and IHC, and further validated by a molecular-based test. Various issues related to the diagnosis of this tumor, as well as the cellular origin of this tumor were discussed.

## Data Availability

Not applicable.

## Abbreviations

FL-HCC: Fibrolamellar hepatocellular carcinoma
IHC: Immunohistochemistry
HCC: Hepatocellular carcinoma
DCP: Des-gamma carboxyprothrombin
CT: Computed tomography
MRI: Magnetic resonance imaging
FNH: Focal nodular hyperplasia
ADC: Apparent diffusion coefficient.
ICC: Intrahepatic cholangiocarcinoma
FFPE: Formalin fixed paraffin embedded
